# Supplementary material for: Conditional chemoconnectomics (cCCTomics) as a strategy for efficient and conditional targeting of chemical transmission
Source: eLife. 2024 Apr 30;12:RP91927. doi: 10.7554/eLife.91927 (PMC11060718; doi:10.7554/eLife.91927)
Supplement: Figure 3—source data 3. [file elife-91927-fig3-data3.zip › Figure 3 source data3 part 2.zip!/Figure 3-Source data 3-part2/1053f3-1-CRISPResso_Report_t6djfp6u.zip!/CRISPResso_on_1053f3_4/9.Alleles_frequency_table_around_sgRNA_ggccaccagatgcaaaatcg.pdf]

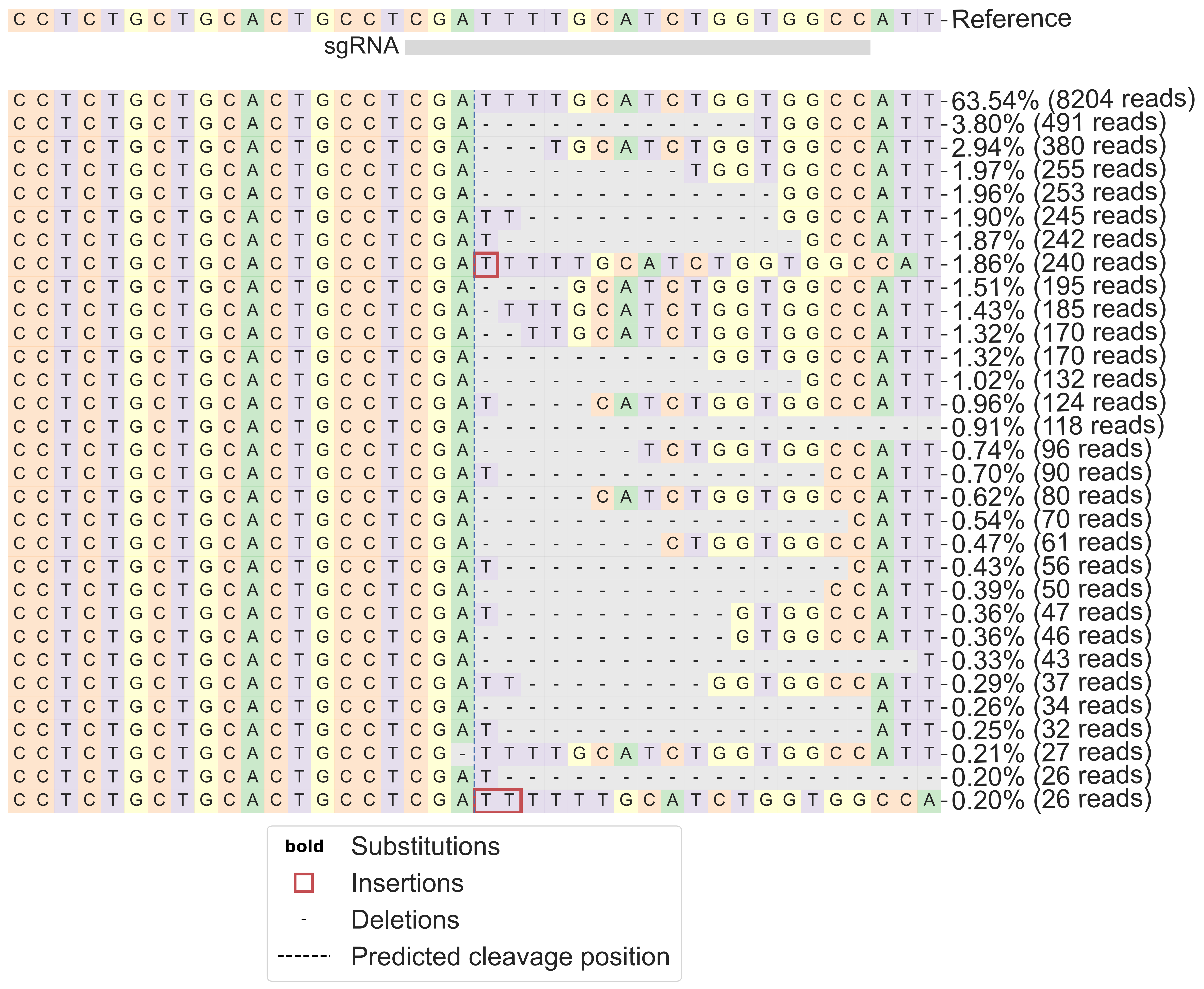
Reference
sgRNA
63.54% (8204 reads)
3.80% (491 reads)
2.94% (380 reads)
1.97% (255 reads)
1.96% (253 reads)
1.90% (245 reads)
1.87% (242 reads)
1.86% (240 reads)
1.51% (195 reads)
1.43% (185 reads)
1.32% (170 reads)
1.32% (170 reads)
1.02% (132 reads)
0.96% (124 reads)
0.91% (118 reads)
0.74% (96 reads)
0.70% (90 reads)
0.62% (80 reads)
0.54% (70 reads)
0.47% (61 reads)
0.43% (56 reads)
0.39% (50 reads)
0.36% (47 reads)
0.36% (46 reads)
0.33% (43 reads)
0.29% (37 reads)
0.26% (34 reads)
0.25% (32 reads)
0.21% (27 reads)
0.20% (26 reads)
0.20% (26 reads)
bold Substitutions
Insertions
Deletions
Predicted cleavage position
